# Satellite DNA landscape in the alpaca genome and comparison with other camelids

**DOI:** 10.1007/s10577-026-09824-3

**Published:** 2026-11-10

**Authors:** Mayra N. Mendoza-Cerna, Hailey C. Anderson, Catherine R. Fox, Edson Aranda, Emily de Anda Pulido, Brian W. Davis, Terje Raudsepp

**Affiliations:** College of Veterinary Medicine and Biomedical Sciences, Texas A&M University, College Station, TX USA

**Keywords:** Heterochromatin, FISH, Tandem repeats, Cytogenetics

## Abstract

Satellite DNAs (satDNAs) are among the least resolved components of telomere-to-telomere (T2T) genome assemblies despite recent advances in long-read sequencing. Among mammals, camelids provide an excellent system to study satDNA evolution because of their exceptional karyotypic conservation despite more than 11 million years of independent evolution. Here, we systematically characterize the satDNA landscape of the chromosome-level alpaca reference genome VicPac4 by combining bioinformatic reference-guided repeat identification, PCR validation, and fluorescence in situ hybridization (FISH). Cross-species PCR analysis of 13 candidate satDNAs revealed two major conservation patterns: six families amplified across all camelids, whereas seven were restricted to South American camelids (SACs). FISH further demonstrated distinct chromosomal organization of nine satDNA families: four showed widespread chromosomal distribution; three localized near centromeres of a subset of chromosomes; one was primarily associated with chromosome 26 but had lower copy number on additional chromosomes, and one exclusively mapped to chromosomes 27 and 36 in all SACs. The latter displayed consistent and prominent copy number differences between species, individuals and homologous chromosomes, suggesting naturally occurring variation in satellite array expansion. This satellite amplified by PCR across Camelidae, whereas FISH detected expanded arrays only in SACs, suggesting differences in satellite organization between SAC and Camels. Beyond satDNA identification, the proposed workflow facilitated the discovery and comparative analysis of pseudogene-rich repetitive blocks in the alpaca genome and their absence in Camels’ genome assemblies. These findings substantially expand the alpaca satDNAs catalogue and provide a cytogenetic framework for future T2T genome refinement and comparative studies across Camelidae.

**Supplementary Information:**

The online version contains supplementary material available at https://doi.org/10.1007/s10577-026-09824-3.

## Introduction

In the era of telomere-to-telomere (T2T) genome assemblies, comprehensive representation of repetitive and heterochromatic regions has become central to understanding genome structure and evolution (Garrido-Ramos 2017; Nurk et al. 2022). Although long-read sequencing and advanced scaffolding strategies have substantially improved assembly contiguity (Rhie et al. 2021), heterochromatic sequences such as satellite DNAs (satDNA) remain among the most challenging parts of the genome to resolve and assign chromosomally, even in well-studied mammalian genomes (Miga & Sullivan 2021; Kalbfleisch et al. 2024).

In this context, Camelids present a particularly intriguing example. The Camelidae family diverged approximately 11–25 million years (MYR) ago (Kadwell et al. 2001; Ji et al. 2009) into two major lineages: the South American camelids (SACs) involving alpaca (*Vicugna pacos*), llama (*Lama glama*), guanaco (*Lama guanicoe*), and vicuña (*Vicugna vicugna*) (Stanley et al. 1994) and the Camels comprised of dromedary (*Camelus dromedarius*), Bactrian camel (*Camelus bactrianus*), and wild camel (*Camelus ferus*) (Stanley et al. 1994; Burger 2016). Despite their long independent evolutionary histories and geographic separation across South America, Africa, and Asia (FAO 2025), all extant species retain the same diploid chromosome number (2 n = 74) (Hungerford & Snyder 1966; Benirschke 1967; Taylor et al. 1968; Hsu & Benirschke 1971; Koulischer et al. 1971). All camelids also exhibit extensive conserved synteny and structural organization of chromosomes (Balmus et al. 2007; Avila et al. 2014). This conserved karyotype likely facilitates interspecific hybridization within each lineage (FAO 2025) and represents a rare evolutionary scenario in which karyotype and chromosome organization has remained stable for over 11 MYR despite ecological and geographic divergence.

Early molecular studies, primarily based on restriction enzyme analyses, provided the first evidence for the presence of satDNA in SACs, revealing both conserved and lineage-specific patterns (Vidal-Rioja et al. 1987). Subsequent comparisons including Camels suggested that some satellite families predate the divergence of Camelidae, whereas others underwent lineage-specific amplification (Vidal-Rioja et al. 1994). However, these approaches did not resolve the precise sequence composition or chromosomal distribution of these repeats, nor did they clarify the organization of the extensive heterochromatic blocks described in camelid chromosomes by cytogenetic studies (reviewed in (Raudsepp et al. 2025)). Using molecular cytogenetic techniques, we recently identified a novel SAC-specific satDNA (SAC-SAT) (Mendoza Cerna et al. 2026), providing direct evidence of lineage-specific heterochromatic differentiation between SAC and Camels.

Here, we expand upon this finding by systematically identifying, characterizing, and chromosomally localizing satDNA in the alpaca genome, the only SAC species with a chromosome-level reference assembly (Mendoza Cerna et al. 2026). We present an assembly-based workflow that integrates genome-wide detection, motif validation, and cytogenetic mapping to assess satellite distribution across individuals and related species. Our results uncover extensive satellite diversity and provide a framework for incorporating highly repetitive sequences into future T2T assemblies and comparative genomic studies of camelids.

## Materials and methods

### Ethics statement

Procurement of samples followed the United States Government Principles for the Utilization and Care of Vertebrate Animals Used in Testing, Research and Training. These protocols were approved as IACUC #2018–0342 CA at Texas A&M University. All methods are in accordance with ARRIVE guidelines (https://arriveguidelines.org) for the reporting of animal experiments.

### In silico identification of satDNA

To investigate the landscape of satDNAin the alpaca genome, we performed the following stepwise computational analysis. Step A. All chromosomes from the VicPac4 alpaca assembly (Mendoza Cerna et al. 2026) were analyzed individually using ModDotPlot (Sweeten et al. 2024) to visualize large-scale tandem repeat organization. Regions displaying strong triangular self-alignment, indicative of high-density tandem arrays, were considered candidate satellite loci. The approximate genomic location of each candidate array was determined from the self-alignment pattern. Step B. These candidate regions were further inspected in Integrative Genomics Viewer (IGV) (Thorvaldsdottir et al. 2013) to evaluate repeat unit organization. The regions identified in step A were examined by zooming into the corresponding genomic intervals in IGV. Step C. Regions were retained when repeat motifs appeared highly enriched and spatially restricted, with minimal representation elsewhere in the genome. Motif-based inspection was used to assess whether the identified motifs showed a tandem repeat pattern. Step D. For each candidate locus, consensus units were retrieved using ULTRA (Olson & Wheeler 2018). Consensus sequences were generated independently and those consistent with the observations from steps A–C were selected. The resulting consensus sequences were further compared against the NCBI nucleotide database using BLASTn to assess similarity to previously characterized sequences. Satellite names were adopted from the nomenclature framework proposed by Ruiz-Ruano and colleagues (Ruiz-Ruano et al. 2016), though the catalog numbers used here serve only as unique identifiers and do not reflect satellite abundance, which was not quantified in this study. Satellites with no suitable motifs were named by species (Vpa) and numerically (Supplementary Table S3).

### Primer design, PCR amplification and probe labeling

Primers flanking portions of each satellite were designed with Primer-BLAST (https://www.ncbi.nlm.nih.gov/tools/primer-blast/). Primer pairs were selected to generate amplicons between 450 and 1500 bp to ensure efficient amplification and suitability for probe generation. Primer specificity was evaluated in silico against the VicPac4 assembly. Genomic DNA (gDNA) from alpaca and other species (Supplementary Table S1) was extracted using the Gentra Puregene Blood kit (Qiagen), following the manufacturer’s protocols. All samples were available at the Texas A&M University Molecular Cytogenetics Laboratory. Initial PCR amplifications for primer optimization and cross-species screening were performed in 10 μL reactions using 5X FIREPol® Master Mix (Solis Biodyne, Estonia). Cycling conditions were as follows: 95 °C (5 min), followed by 40 cycles of 95 °C (30 s), 60 °C (40 s), and 72 °C (1 min), with a final extension at 72 °C for 10 min. PCR products and a 100 bp DNA ladder (New England Biolabs) were analyzed by electrophoresis on 2% agarose gels stained with ethidium bromide and visualized using a UVP BioDoc-It Imaging System. Primer pairs that yielded clear and reproducible amplification products were selected for probe generation. For this, PCR reactions were scaled up to 100 μL under the same cycling conditions. Amplified products were purified using the QIAquick PCR Purification Kit (Qiagen) and labeled by nick translation using either Biotin-Nick or DIG-Nick Translation Mix (Roche Diagnostics), following the manufacturer’s protocol. All primer sequences are listed in Table 1.

**Table 1:** Primer sequences and expected amplicon sizes of satDNA

Satellite name	Primer name	Forward primer 5’−3’	Reverse primer 5’−3’	Product size, bp
VpaSat01–3503	VpaSat01–3503-primer2	TCAATGAGTGGTGCTGGGAA	TCTGCATGTCCTCTTTGGGG	465
VpaSat02–5021	VpaSat02–5021-primer1	CAGACATGGGTGTTCTGGGG	CGGGAAACGCAAAGATACGC	675
VpaSat03–3224	VpaSat03–3224-primer1	TCCTCCCCTGAGAGACAGAG	TAGCCTATGTGTGTGCCCATC	514
VpaSat04–5242	VpaSat04–5242-primer1	CGGACGCTAGAAAGGGACAC	TCAGCCTGGTGGCTAACTTG	1065
VpaSat05–8550	VpaSat05–8550-primer2	AGTAGGGTGCAGGCCCATGTTA	TGAGCTGACCCTGTCCTTGA	511
VpaSat06–n/a	VpaSat06–n/a-primer1	CCCTGTGTTACCTTTCGGTCC	GGGCCTTCCTCCTAAATGGC	1651
VpaSat07–n/a	VpaSat07–n/a-primer3	CACTTTTCTCGCGGAAGCTC	GCCTCAGTTTTCCGTCCACT	837
VpaSat08–5290	VpaSat08–5290-primer1	GTGGAGCTTGGCTTAGGTGT	GCTAGCTAGGCCCAAACCTG	603
VpaSat09–5280	VpaSat09–5280-primer1	TCACAGGTCGGTTTGCTCTC	CTCACTCGCTCCCATGAACA	493
VpaSat10–3126	VpaSat10–3126-primer1	CTGTAGGCTAGTGTGCCGTT	ACACACTGGCGTCCTACTTC	623
VpaSat11–5308	VpaSat11–5308-primer1	GCACAGAACACTTGCCGAAG	AATGCCAAATTGGTCCCTGG	543
VpaSat12–5280	VpaSat12–5280-primer1	GGACACACACTCTCAAGCGA	GATCCCACAGACCGACCTTG	734
VpaSat13–2326	VpaSat13–2326-primer2	TTGGGTTTCGGCTTTTGTCG	CACAGGACACACAGTCTGGAG	905

### Metaphase chromosome preparations

Metaphase chromosome preparations were obtained from primary fibroblast cultures or stimulated peripheral blood lymphocyte cultures of alpaca, llama, guanaco, vicugna, dromedary, and Bactrian camel (Supplementary Table S1) using established protocols (Raudsepp & Chowdhary 2008). Briefly, fibroblasts were cultured in alpha MEM (Gibco) supplemented with 20% FBS (Taurus Biosciences, Houston, Teas) and 1 × Antibiotic–Antimycotic (Anti-Anti 100x, Gibco) at 37 °C and 5% CO_2_. For blood lymphocyte cultures, 1 mL of sodium heparin stabilized whole blood was grown for 72 h in 9 mL of culture medium containing RPMI-1640 with HEPES and Glutamax (Gibco), 30% fetal bovine serum, 1 × Antibiotic–Antimycotic, and 15 μg/mL Concanavalin A (Sigma Aldrich). Fibroblasts and lymphocytes were harvested with KaryoMAX™ Colcemid™ Solution (Gibco; final conc. 0.1 μg/mL), treated with Optimal Hypotonic Solution (Rainbow Scientific) for 15 min at 37 °C, and fixed in 3:1 methanol/acetic acid. The cells were dropped on pre-cleaned wet glass slides and air dried. The quality of chromosome preparations was examined under a light microscope with a × 20 phase contrast objective. All samples were procured through the Texas A&M University Molecular Cytogenetics Laboratory chromosome analysis service.

### Fluorescence in situ Hybridization (FISH) and analysis

Hybridizations were conducted following standard protocols (Raudsepp & Chowdhary 2008). Biotin-labeled and digoxigenin-labeled probes were detected with avidin-FITC (Vector Laboratories) and anti-digoxigenin-rhodamine (Roche Diagnostics), respectively*.* FISH slides were mounted in ProLong™ Gold Antifade with DAPI (Invitrogen) and analyzed with an Axio Imager M2p microscope (Carl Zeiss, Inc., Jena, Germany) equipped with a high-resolution progressive scan CCD camera CoolCube1 and ISIS V5.2 software (MetaSystems GmbH, Altlussheim, Germany). Multiple dual-color FISH experiments were performed combining satDNA probes with probes specific to different alpaca autosomes (Avila et al. 2014; Mendoza et al. 2019, 2020; More et al. 2019) (Supplementary Table S2). For each FISH experiment at least 10 representative images were captured and analyzed.

## Results

### Genome-wide identification of satDNA in alpaca

**Fig. 1:**
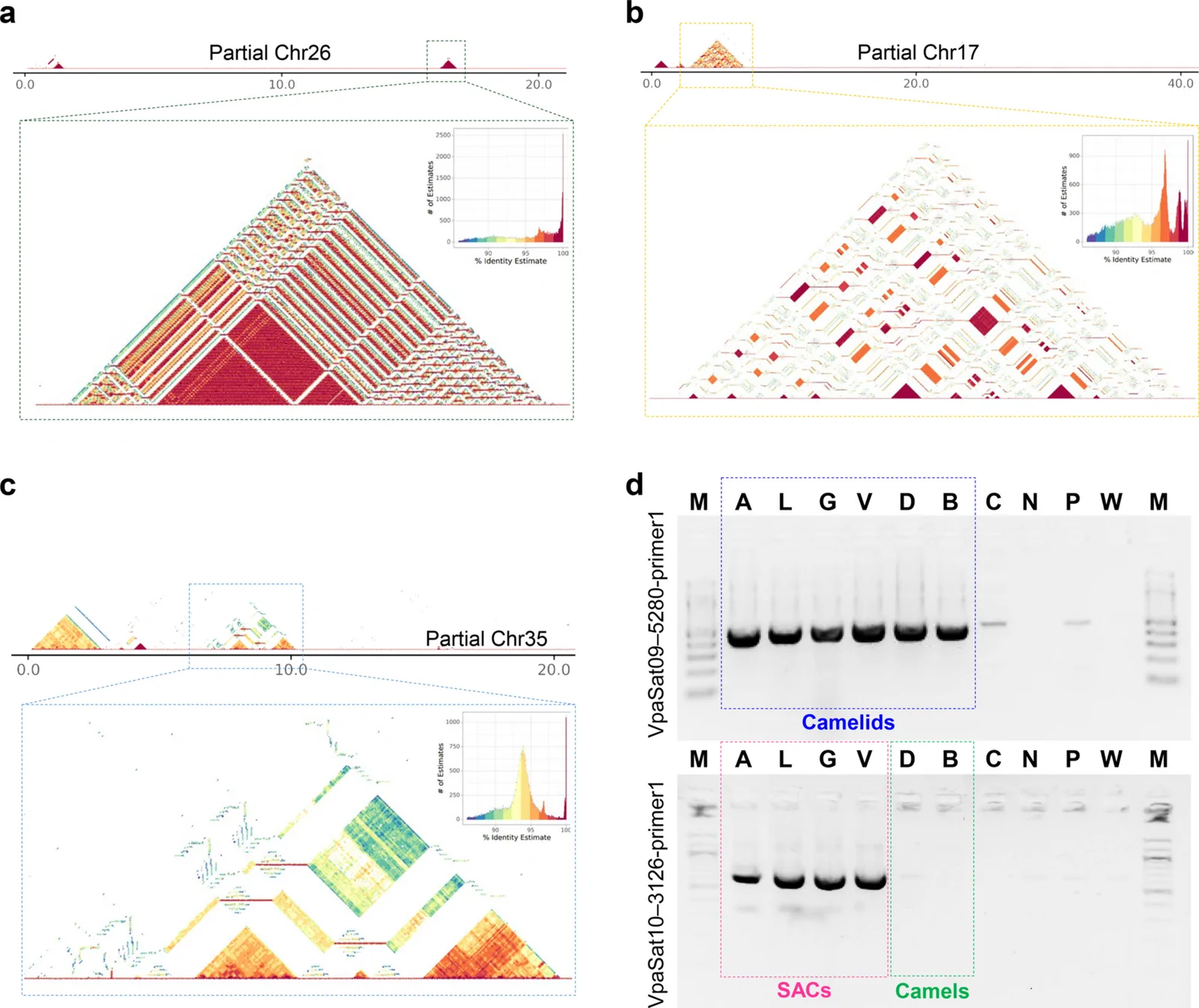
Genome-wide identification and validation of satellite DNA in alpaca. (**a**) Dotplot (green inset) of part of chromosome 26 showing a strong and continuous triangular self-alignment pattern; (**b**) Dotplot (yellow inset) of part of chromosome 17 showing a discontinuous triangular self-alignment pattern; (**c**) Dotplot (blue inset) of part of chromosome 35 showing a diffuse triangular pattern characteristic of satDNA associated with centromeric regions; (**d**) Validation of satDNA by PCR; Top: VpaSat09–5280-primer1 is present in all camelids; Bottom: VpaSat10–3126-primer1 is present only in SAC; M – 100 bp ladder; A—alpaca; L—llama; G—guanaco; V—vicuña; D—dromedary; B—Bactrian camel; C—cattle; N—Soemmerring’s gazelle; P—pig; W—water

Genome-wide generation of sequence similarity plots of each chromosome in the VicPac4 assembly revealed multiple regions with triangular self-alignment patterns indicative of tandem repeat organization. These loci were broadly classified into three categories based on the structure of self-alignment: (i) 17 loci displayed strong and continuous triangular patterns, consistent with homogeneous tandem arrays typical of canonical satDNA (Fig. 1a; Supplementary Figure S1); (ii) three regions showed discontinuous self-alignment patterns, indicative of interrupted repeat arrays (Fig. 1b; Supplementary Figure S1), and (iii) a subset of loci showed diffuse triangular patterns associated with centromeric regions (Fig. 1c; Supplementary Figure S1). In total, 20 non-centromeric candidate satellite loci were identified in 13 chromosomes (Supplementary Figure S1). Of these, consensus monomer sequences were identified for 14 satDNA (Supplementary Table S3) with repeat unit length ranging from ~ 1.8 kb to ~ 8.5 kb (Table 2). Consensus sequences could not be reconstructed for the remaining six loci. BLASTn searches of the 14 consensus sequences did not identify significant matches to previously characterized satDNA, although, several consensus sequences showed similarity to genomic sequences from camelid species.

**Table 2:** Sequence characteristics, chromosomal localization and chromosomal distribution of satDNA families in the alpaca genome

Satellite name	Motif size, bp	AT %	Chromosomes	Location	GenBank Accession
VpaSat01–3503	3503	69.5	n/a	n/a	QB059667
VpaSat02–5021	5021	45.2	8–10 pairs	below centromere	QB059668
VpaSat03–3224	3224	43.2	8–10 pairs	below centromere	QB059669
VpaSat04–5242	5242	28.2	predominantly Chr26	interstitial	QB059670
VpaSat05–8550	8550	36.5	n/a	n/a	QB059671
VpaSat06–n/a	n/a	n/a	n/a	n/a	n/a
VpaSat07–n/a	n/a	n/a	n/a	n/a	n/a
VpaSat08–5290	5290	54.9	16–20 pairs	above centromere	QB059672
VpaSat09–5280	5280	54.3	16–20 pairs	above centromere	QB059673
VpaSat10–3126	3126	45.4	8–10 pairs	below centromere	QB059674
VpaSat11–5308	5308	54.2	16–20 pairs	above centromere	QB059675
VpaSat12–5280	5280	55.6	16–20 pairs	above centromere	QB05967
VpaSat13–2326	2326	36.6	Chr27 and Chr36	interstitial	QB059677

### PCR validation, cross-species amplification and probe generation

Primers could be designed for 13 out of the 20 candidate satellites (Table 1), although the loci with successful primer design only partially overlapped with those for which consensus monomer sequences were reconstructed (Supplementary Table S3). Primer design was not possible for the remaining loci, due to low sequence complexity and the absence of suitable unique regions for specific amplification. Three of those were retained as putative satellites based on in silico validation of their consensus sequences. All 13 primer pairs successfully amplified from alpaca gDNA, generating products with the expected size (Table 1). Next, the primers were used for cross-species amplification to assess the conservation of satDNA across camelids and select non-camelid mammals. Six satellites (VpaSat01–3503, VpaSat08–5290, VpaSat09–5280, VpaSat11–5308, VpaSat12–5280 and VpaSat13–2326) showed consistent amplification in all tested camelid species, whereas seven satellites (VpaSat02–5021, VpaSat03–3224, VpaSat04–5242, VpaSat05–8550, VpaSat06–n/a, VpaSat07–n/a and VpaSat10–3126) were detected exclusively in SACs (Fig. 1d; Supplementary Figure S2).

VpaSat06–n/a failed to amplify in vicuña. However, because the expected amplicon size was relatively large (1,651 bp), unsuccessful amplification was likely due to low gDNA quality (fragmentation) rather than the absence of the target sequence. In addition, VpaSat12–5280 showed two amplicons in the dromedary (Supplementary Figure S2). The majority of the tested satDNAs showed no evidence of sequence conservation outside Camelidae and, thus, no amplification by PCR. VpaSat01–3503 was the only satellite that consistently amplified in non-camelid species, whereas VpaSat02–5021, VpaSat03–3224 and VpaSat08–5290 produced non-specific amplification with different banding patterns in non-camelids.

Primers of all 13 satDNAs that successfully amplified in the alpaca were subsequently used to generate probes for FISH.

### Chromosomal localization of satDNA

**Fig. 2:**
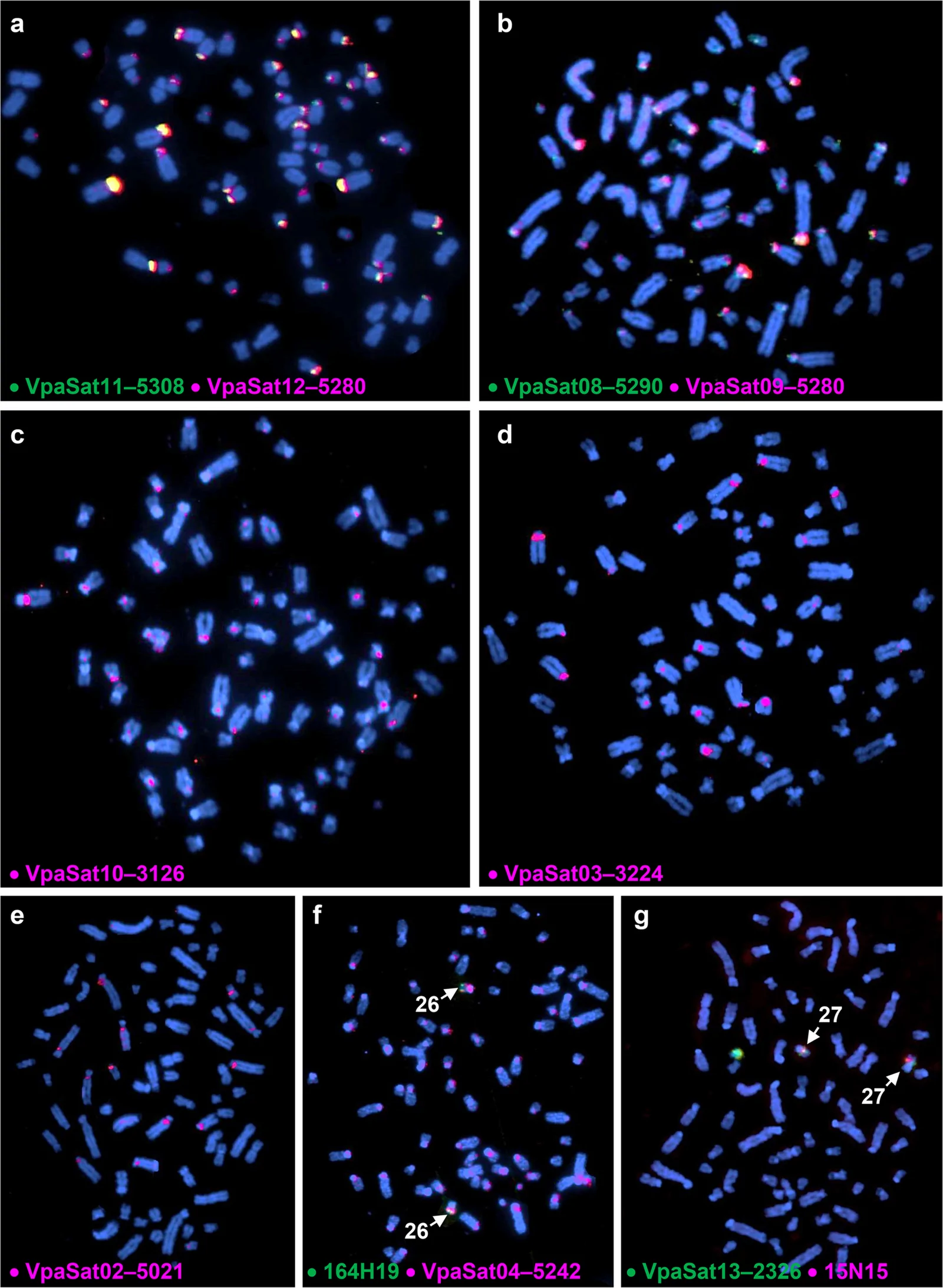
Cytogenetic localization of selected satDNA families in alpaca by FISH. (**a**) VpaSat11–5308 (green) and VpaSat12–5280 (red) co-localize above the centromere in multiple chromosomes; (**b**) VpaSat08–5290 (green) and VpaSat09–5300 (red) co-localize above the centromere in multiple chromosomes; (**c**) VpaSat10–3126 (red) maps below the centromere in approximately 8–10 chromosome pairs; (**d**) VpaSat03–3224 (red) maps below the centromere in 8–10 chromosome pairs; (**e**) VpaSat02–5021 (red) maps below the centromere in 8–10 chromosome pairs; (**f**) VpaSat04–5242 (red) maps predominantly to chromosome 26 (BAC 164H19, green; arrows). (**g**) Mapping VpaSat13–2326 (green) to chromosome 27 (BAC 15N15, red; arrows); Chromosomes are counterstained with DAPI (blue)

Fluorescence in situ hybridization revealed diverse chromosomal distribution patterns of satDNA families, ranging from chromosome-specific location to widespread presence across multiple chromosomes (Table 2). Most satellites were located at pericentromeric regions, although several interstitial sites were also detected (Fig. 2; Table 2). Satellites VpaSat08–5290, VpaSat09–5280, VpaSat11–5308 and VpaSat12–5280, were initially identified as four independent satellites based on in silico analysis. However, FISH revealed that both pairs of satellites are widely distributed across many chromosomes (16 to 20 pairs), with strong enrichment in the short arms above the centromeric regions (Fig. 2a, b). Co-localization of these satellites was also observed in llama, guanaco, and vicugna (Supplementary Figure S3), suggesting substantial sequence homology, consistent with the pairwise sequence comparisons between VpaSat11–5308 and VpaSat12–5280 and between VpaSat08–5290 and VpaSat09–5280, but also between VpaSat11–5308 and VpaSat08–5290, and between VpaSat12–5280 and VpaSat09–5280. Dotplot analyses showed extended collinear regions shared among these satellites, although pairwise identity varied from approximately 25% to 50% (Supplementary Figure S4). The results indicate that these satellites represent a major, widely dispersed satellite family in the alpaca genome.

Satellites VpaSat02–5021, VpaSat03–3224, and VpaSat10–3126 were detected in fewer chromosomes (8–10 pairs) and were consistently localized in the long arm below the centromere (Fig. 2c-e).

**Fig. 3:**
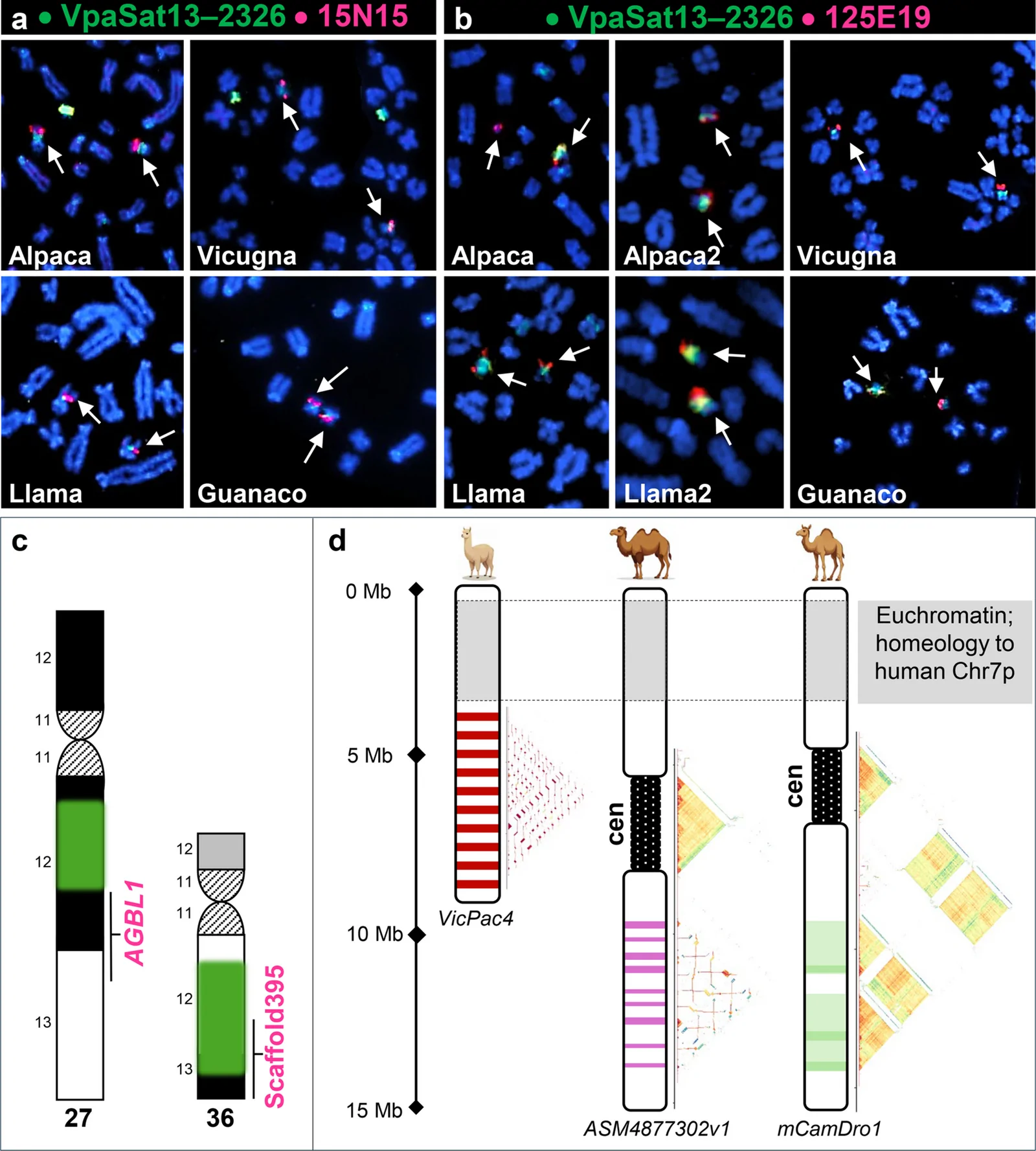
Localization and comparative organization of VpaSat13–2326 in camelids; (**a**) FISH with VpaSat13–2326 (green) and BAC 15N15 (chromosome 27 marker, red) in SAC; (**b**) FISH with VpaSat13–2326 (green) and BAC 125E19 (chromosome 36 marker, red) in SAC; note VpaSat13–2326 copy number differences between chromosome 36 homologs; (**c**) Schematic drawings of alpaca chromosomes 27 and 36 showing cytogenetic band nomenclature (left) and the location of chromosome-specific FISH markers (right) and VpaSat13–2326 (green shade); (**d**) Dotplot-based comparison of chromosome 36 assemblies between alpaca, Bactrian camel, and dromedary; the gene-rich euchromatic region with conserved synteny with human chromosome 7p is highlighted in grey; VpaSat13–2326 is denoted with horizontal red, purple and green lines, respectively; dotplots corresponding to VpaSat13–2326 in the three species and to Bactrian camel and dromedary centromeres (cen) are to the right

In contrast, VpaSat04–5242 showed predominant localization in chromosome 26, consistent with in silico predictions, although weaker hybridization signals were also detected across several other chromosomes. VpaSat13–2326 distinctly localized interstitially in two chromosome pairs (Fig. 2g). Dual-color FISH using chromosome-specific probes revealed that in SACs, VpaSat13–2326 localizes to chromosomes 27 and 36, and shows consistent copy number differences between chromosome 36 homologs (Fig. 3a-c). Interestingly, PCR amplification with VpaSat13–2326 primers confirmed the presence of this sequence in all camelids (Supplementary Figure S2), however no detectable FISH signal was observed in Camels using alpaca-derived probe. This may be due to differences in VpaSat13–2326 repeat organization between SAC and Camels as revealed by comparative analysis of chromosome 36 assemblies from alpaca, Bactrian camel, and dromedary, together with their corresponding dotplot profiles (Fig. 3d).

Finally, not all candidate satellites yielded informative FISH results. VpaSat01–3503 did not produce detectable hybridization signals, while probes for VpaSat05–8550, VpaSat06–n/a and VpaSat07–n/a mapped to all centromeres. Such signals may reflect true enrichment of these satDNAs in centromeric regions, although non-specific hybridization or probe-related artifacts cannot be excluded. Because the present study focused on the characterization of non-centromeric satDNAs, these probes were not used for further FISH analyses.

### Identification of pseudogene-rich satDNA blocks

**Fig. 4:**
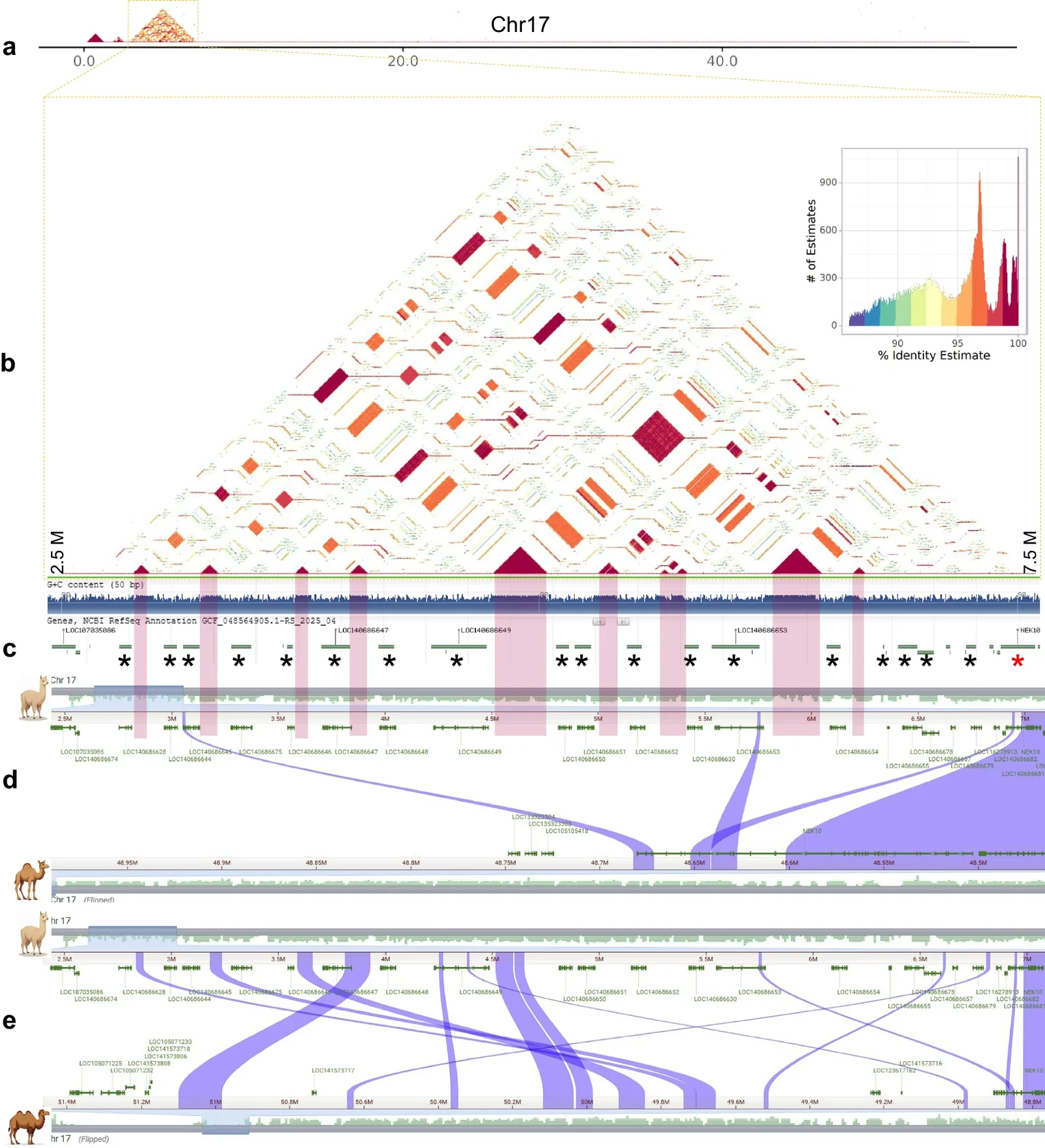
Repetitive pseudogene-rich block in chromosome 17. (**a**) Dotplot of chromosome 17; (**b**) Enlarged dotplot (yellow inset) of a ~ 4 Mb discontinuous satDNA spanning ~ 2.5–7.5 Mb; (**c**) Annotation track in VicPac4 showing that regions intervening high-identity triangular dotplots (pink shades) are enriched with 18 copies of Nek10-like sequences (*); the actual *NEK10* gene (red * most right) is outside the repetitive block; (**d**) Comparison of this region between alpaca VicPac4 and dromedary *mCamDro1.pat*, and (**e**) Comparison of this region between alpaca VicPac4 and Bactrian camel *ASM4877302v1*

In contrast to the normal continuous self-alignment patterns of repetitive regions in most chromosomes, prominent clusters of repeats in alpaca chromosomes 11, 17 and 36, showed discontinuous self-alignment (Figs. 1b, 3d, 4a; Supplementary Figures S5, S6). Detailed inspection of a region from 2.5 Mb to 7.5 Mb in chromosome 17 (Fig. 4b) revealed the presence of multiple serine/threonine-protein kinase Nek10 (*NEK10*)-like sequences distributed across a ~ 4 Mb interval. In total, 19 Nek10-related sequences were identified in the VicPac4 assembly, including one annotated *NEK10* gene and multiple Nek10-like copies of varying length and completeness. These copies are arranged in a dispersed manner within a repeat-rich genomic landscape and do not form a simple tandem array. Integration of dotplot patterns with sequence annotation showed that the high-identity triangular signals correspond to regions with elevated sequence similarity and GC content, whereas the intervening regions contain most of the Nek10-like fragments (Fig. 4c). Comparative analysis using *mCamDro1.pat* dromedary genome and *ASM4877302v1* Bactrian camel genome, revealed a single-copy *NEK10* locus in Camels, with no evidence of the expanded multi-copy structure like observed in alpaca (Fig. 4d-e).

A similar but larger repetitive region was identified on chromosome 11 (Supplementary Figure S5). This region spans ~ 10 Mb and contains multiple copies of gene-related sequences, including ~ 12 copies of cyclic AMP-dependent transcription factor ATF-5 pseudogene elements and ~ 10 copies of formin-2-like (*FMN2*-related) sequences. These elements are also arranged as dispersed and fragmented copies of variable length.

Similarly, on chromosome 36 (Supplementary Figure S6), multiple gene-related fragments are embedded within a repeat-rich domain. These include five copies of small ribosomal subunit protein uS3m-like sequences, seven copies of actin cytoplasmic 1 pseudogenes and fifteen copies of alpha/beta hydrolase domain-containing protein 17 A-like elements. Notably, the latter gene family is also represented by three copies on chromosome 27. No comparable repetitive regions corresponding to the expanded portions on alpaca chromosomes 11 and 36 were detected in dromedary or Bactrian camel genomes.

## Discussion

Identification and chromosomal assignment of nine new satDNA families in the alpaca represents the first systematic, chromosome-level characterization of repetitive DNA in Camelids. These satDNAs were derived from an initial set of 20 candidate loci, followed by motif characterization for 14 sequences (Supplementary Table S3), subsequent PCR-based validation of 13 satellites (Table 1) of which nine were assigned to chromosomes by FISH (Table 2). Aside from the recent identification of SAC-SAT, a satDNA associated with nucleolus organizing regions (NORs) in SAC species (Mendoza Cerna et al. 2026), no comprehensive studies on satellite DNA diversity have been done in Camelids. In contrast, satDNA has been characterized in diverse mammalian lineages (Alkan et al. 2011), including humans (Altemose et al. 2022), primates (Alkan et al. 2007; Araújo et al. 2017), rodents (Pardue & Gall 1970; Vissel & Choo 1989), felids (Fanning 1987; Harris et al. 2025), bats (Deon et al. 2025), suiforms (Adega et al. 2008), bovids (Nieddu et al. 2015; Escudeiro et al. 2019) and cervids (Vozdova et al. 2020). Here, we address this gap in knowledge by providing the first integrated analysis of satDNA in alpaca at whole genome sequence and cytogenetic levels.

Notably, VpaSat13–2326 had restricted chromosomal distribution, confined to chromosomes 27 and 36 (Fig. 3), thus deviating from the traditional view of satDNA as large repetitive arrays broadly clustered within heterochromatic regions all over the genome (Satovic-Vuksic & Plohl 2023). Although less common than multi-chromosomal satellites, similar restricted satDNA patterns have been reported also in some other species, particularly in association with sex chromosomes or supernumerary (B) chromosomes (Ugarkovic & Plohl 2002; Plohl et al. 2012; Pita et al. 2017; Montiel et al. 2022). Such distinct distribution patterns are consistent with the concept of concerted evolution, in which satellite arrays are homogenized within a chromosome but do not necessarily spread efficiently between chromosomes (Dover 1986). It is also noteworthy that this chromosomally confined satellite was interstitial rather than pericentromeric. This positional difference may contribute to restricted distribution, as interstitial loci are less frequently associated with the large-scale homogenization and amplification processes typically observed in centromeric satellite arrays. Similar observations have been reported in other species where non-centromeric satellites exhibit more localized or chromosome-specific patterns (Ugarkovic & Plohl 2002; Plohl et al. 2012; Pita et al. 2017; Montiel et al. 2022).

In contrast, seven satellite families (VpaSat02–5021, VpaSat03–3224, VpaSat08–5290, VpaSat09–5280, VpaSat10–3126, VpaSat11–5308 and VpaSat12–5280) were located near the centromere in multiple chromosomes (Figs. 2a-e). Similar distribution patterns have been described in other mammals, where satellite families are often shared between non-homologous chromosomes but maintain conserved positional biases, particularly in pericentromeric or sub telomeric regions (Charlesworth et al. 1994; Plohl et al. 2012). These observations support a non-random organization of satDNA into distinct chromosomal compartments, a feature widely associated with differences in sequence composition, chromatin state, and evolutionary dynamics of repetitive DNA (Ugarkovic & Plohl 2002). Among these, VpaSat08–5290, VpaSat09–5280, VpaSat11–5308 and VpaSat12–5280, represent prominent satellite families in alpaca, with widespread distribution in multiple chromosomes (Fig. 2a). Interestingly, comparative analysis of VpaSat11–5308 and VpaSat09–5280 in llama, guanaco and vicugna indicated the same distribution pattern (Supplementary Figure S3). Similar chromosomal location of these satellites in all SAC species may just reflect the retention of ancestral chromosomal arrangements or conserved genomic regions but may be also associated with special features of satDNA evolution, known as the “library hypothesis”. This hypothesis suggests that related species share a common set of satellite sequences that undergo differential amplification or contraction over time (Fry & Salser 1977; Garrido-Ramos et al. 2025). “Library hypothesis” could also explain why seven satDNA families were restricted to SAC, whereas six satDNA families amplified across the entire Camelidae. Comparative analysis of satellite abundance and sequence divergence between the species would be necessary to establish a stronger connection between the observed distribution patterns and the library model.

It is important to note that many satDNA families were in silico identified only in certain chromosomes (Supplementary Figure S1) but were subsequently detected in multiple chromosomes by FISH. This discrepancy reflects the incomplete representation of highly repetitive regions in non-T2T assembled chromosomes, particularly in pericentromeric regions where large satellite arrays are typically collapsed or absent by bioinformatic analysis. This limitation has been widely documented in genome assemblies, where repetitive DNA remains one of the most challenging components to resolve (Logsdon et al. 2021; Miga & Sullivan 2021). Thus, our workflow provides a practical starting point for the characterization and chromosomal validation of satDNA even in the absence of a T2T assembly, which remains unavailable for many species. Complementary read-based approaches could subsequently help identify additional satellite families, particularly those that are smaller or poorly represented in the current assembly.

In addition to canonical satDNA, we identified large pseudogene-rich repetitive blocks intermingled with discontinuous satDNA, providing further insight into the dynamics of camelid genomes. Here the region in chromosome 11 represents a striking example of lineage-specific genomic remodeling (Supplementary Figure S5). The presence of multiple *ATF-5* pseudogene elements and fragmented *FMN2*-like sequences within a ~ 10 Mb interval suggests that this expansion is driven by localized duplication events rather than uniform repeat accumulation. Comparative analysis with the Camel genomes indicates that this region is absent or substantially reduced which could be due to incomplete representation of repetitive regions in non-T2T genomes. However, earlier cytogenetic observations that guanaco chromosome 11 is distinctly larger than its dromedary counterpart (Balmus et al. 2007), provide independent, though indirect, support for lineage-specific expansion of these sequences in alpaca and other SAC. Although a chromosome-level assembly is not available for guanaco, its close evolutionary relationship with alpaca justifies the use of the alpaca genome as a representative model for SACs. Under this framework, the expanded region identified on alpaca chromosome 11 (Supplementary Figure S5) provides a plausible molecular basis for the previously reported size difference between guanaco and dromedary homeologs (Balmus et al. 2007). We theorize that similar duplication-driven expansions may be shared across SAC genomes, thus underscoring the need for high-quality, representative genome assemblies for all camelid species.

A similar pattern, albeit at a smaller scale, was observed in chromosomes 17 and 36, which contain expanded regions of ~ 4 Mb (Fig. 4b) and ~ 5 Mb (Supplementary Figure S6), respectively. As in chromosome 11, these multicopy pseudogene-like sequences are absent or substantially reduced in camel homeologs. Although the gene-derived sequences identified in these regions originate from functionally diverse loci (*NEK10* – cell cycle regulation; *uS3m –* ribosomal translation; *ACTB –* cytoskeleton; *ABHD17A* – membrane-associated signaling*)*, they share common structural features, including fragmentation, multi-copy organization, and localization within repeat-rich domains. This pattern suggests that their accumulation is primarily driven by genomic context rather than functional selection. These observations are consistent with the enrichment of duplicated sequences in genomic regions of reduced recombination near centromeric and/or repeat-rich domains (Charlesworth et al. 1994). They are also compatible with segmental duplication followed by sequence degeneration, a process known to generate structurally complex genomic compartments in mammalian genomes (Bailey & Eichler 2006; Marques-Bonet et al. 2009). These mechanisms are not mutually exclusive and may jointly contribute to the observed architecture. Although pseudogenization typically results in loss of coding capacity, duplicated gene fragments can retain or acquire regulatory roles, including transcriptional and post-transcriptional modulation (Poliseno et al. 2010; Pink et al. 2011). In this context, the pseudogene-rich satDNA in the alpaca and other SAC genomes are of potential functional interest.

Previous studies have repeatedly pointed at the smallest camelid autosome, chromosome 36, as the most challenging chromosome in these genomes. This cytogenetically very heterochromatic chromosome failed to reveal human homeology in the initial human-dromedary Zoo-FISH (Balmus et al. 2007) and assembly of chromosome 36 has stayed the genome poorest in both VicPac3 (Richardson et al. 2019) and VicPac4 (Mendoza Cerna et al. 2026). Analysis of chromosome 36 assemblies in available camelid genomes also reveals marked structural difference between species. In alpaca, this chromosome spans ~ 9 Mb, whereas in both Bactrian camel and dromedary it reaches ~ 15 Mb, including a centromeric region of 3–4 Mb. Notably, the only gene-rich euchromatic segment that shares conserved synteny with part of human chromosome 7p, is restricted only to ~ 3–4 Mb (Fig. 3d), while the rest of the alpaca chromosome is comprised of the complex sequence of VpaSat13–2326 which explains assembly difficulties. In SACs, VpaSat13–2326 is also present in chromosome 27 as revealed by FISH (Fig. 3a, b).

In camels, VpaSat13–2326 was detected by PCR (Supplementary Figure S2), but not by FISH. This discrepancy is supported by the in silico PCR using VpaSat13–2326 primers (expected product size 905 bp, Table 1) across available camelid genome assemblies. The analysis revealed over 200 predicted amplicons (904–906 bp) in alpaca (VicPac4), all mapping to chromosomes 27 and 36. In contrast, only 23 and 33 amplicons (925–936 bp) were detected in dromedary (mCamDro1) and Bactrian camel (ASM4877302v1) assemblies, respectively, distributed across multiple chromosomes and unassigned scaffolds. Together, the lower copy number, dispersed genomic distribution, and greater sequence divergence of VpaSat13–2326 in camels may explain the absence of detectable FISH signals despite successful PCR amplification. These findings underscore the complementary value of FISH to sequence-based analyses for resolving the chromosomal organization of satDNA. Additionally, dotplot analysis showed that the non-genic portion of dromedary and Bactrian camel chromosome 36 comprises satellites of different repeat structures, whereas these are different even between the two camel species (Fig. 3d), suggesting distinct lineage-specific repetitive expansions.

In SACs, the relative abundance of VpaSat13–2326 sequence considerably varies between species, individuals and even homologous chromosomes as revealed by the intensity of FISH signals (Fig. 3b). This inter- and intra-individual variability suggests that VpaSat13–2326 is under ongoing cycles of expansion and contraction, consistent with the dynamic satellite DNA evolution (Fry & Salser 1977; Garrido-Ramos et al. 2025). Besides the observed VpaSat13–2326-derived polymorphism in normal individuals, notable size difference between chromosome 36 homologs is a characteristic feature of the Minute Chromosome Syndrome (MCS) which is associated with underdeveloped reproductive tract and infertility in female alpacas (Avila et al. 2015). In MCS, however, the main cause of size difference is the emergence of an ectopic rDNA cluster (NOR) in one chromosome 36 homolog, so that the normal chromosome appears much smaller. On the other hand, we recently showed that NORs in South American Camelids, are associated with a specific satellite, SAC-SAT (Mendoza Cerna et al. 2026). Sequence motifs and chromosomal distributions of SAC-SAT and VpaSat13–2326 are different and because alpacas do not have NOR in chromosome 36, the two satellites are normally not co-localizing in this chromosome. However, because of the ectopic NOR in alpacas with MCS, the larger, abnormal chromosome 36 also carries SAC-SAT. Collectively, this implies that the observed size polymorphism of chromosome 36 in MCS is driven not only by ectopic NOR but also by the abundance of SAC-SAT and VpaSat13–2326. It is known that tandemly repetitive nature of rDNA and satDNA poses challenges for DNA replication and repair and can lead to DNA breaks and rearrangements and chromosome-level instability (Flynn & Yamashita 2024). However, whether there is a link between the “excess” of repetitive DNA in chromosome 36 and the infertility phenotype of MCS, remains for now speculative and enigmatic.

Finally, there are notable similarities between chromosomes 27 and 36 repeat content. Both carry the discontinuous VpaSat13–2326 intermingled with duplicated *ABHD17A*-like elements, and both are NOR/SAC-SAT chromosomes in most camelids (except chromosome 36 in alpaca) (Mendoza Cerna et al. 2026). Chromosome 27 also contains the *SNORD116* cluster, composed of multiple tandem repeats downstream of the *SNURF-SNRPN* locus, a region orthologous to the imprinted domain on human chromosome 15q11–q13 (Good & Kocher 2017). Together, these features suggest that chromosomes 27 and 36 may represent genomic compartments permissive to repeat accumulation.

Taken together, besides its relevance to alpaca genome organization, this work provides a framework for the analysis of satDNA in future T2T assemblies. As more complete genomes become available for SACs, integration of genome-based analyses with molecular and cytogenetic validation will be essential to resolve the full extent and organization of repetitive DNA. 

## Supplementary Information

Supplementary Material 1.

Supplementary Material 2.

## Data Availability

Consensus sequences of the satellite DNA families identified in this study have been deposited in GenBank under accession numbers QB059667–QB059680. All other data are available in the main text and in Supplementary materials.
